# 4D-flow assessment of cerebral hemodynamic in patients with post EC-IC bypass

**DOI:** 10.1186/1532-429X-14-S1-W40

**Published:** 2012-02-01

**Authors:** Tetsuro Sekine, Yasuo Amano, Ryo Takagi, Yoshio Matsumura, Yuriko Suzuki, Shinichiro Kumita

**Affiliations:** 1 Radiology, Nippon Medical School, Bunkyo-ku Sendagi, Japan; 2 Philips Electronics Japan, Tokyo, Japan

## Summary

The aim of this study was to visualize the complicated blood flow of the cerebral arteries in patients with post extracranial-intracranial (EC-IC) bypass using time-resolved three dimensional phase-contrast (4D-Flow) MRI.

## Background

EC-IC bypass surgery is performed in the patients with internal carotid artery occlusion (ICO) or who undergo ligature of the internal carotid artery (ICA) for the treatment of aneurysm. The flow directions are varied according to the relative pressure of some intracranial arteries following post EC-IC bypass. Abnormal retrograde flow is often observed at the ipsilateral M1 segment of the middle cerebral artery (MCA), circles of Willis and ophthalmic artery (OphA) to compensate the decrease of flow from ICA. 4D-Flow can be a valuable tool for visualizing these complicated flows with unrestricted view, non-invasiveness, and high reproducibility.

## Methods

19 patients (12 men; mean age, 64 years), including 10 with ICO and 9 with post ligation of ICA, were investigated. The EC-IC bypass was successfully achieved and clinical status was improved in all patients. MR studies were performed on a 3T unit (Philips). The imaging parameters of 4D-Flow MRI were as follows; TR/TE=8.4/5.4, FA=13, FOV=210X210mm, VENC=70cm/sec, voxel size=0.82X0.82X1.4mm, heart phase= 15. 4D Flow -streamline images of the cerebral arteries were generated using the GTFlow software (GyroTools). The patency of donor artery was assessed. The direction of flow at M1 segment of middle cerebral artery, A1 segment of anterior cerebral artery, posterior communicating artery (PCoA) and OphA were assessed in the ipsilateral side. The direction of each flow was categorized into retrograde, anterograde and unclear (very slow or no flow).

## Results

In all 19 patients, 4D-Flow MRI successfully visualized the whole cerebral arteries. The patency of donor artery was proved in all cases. Abnormally retrograde flow was observed at M1 (n = 9: 47.4%), A1 (n=5: 26.3%), PCoA (n=13: 68.4%), and OphA (n=6: 31.6%). The retrograde flow was observed more frequently at M1 in the patients with post ligation of ICA than in those with ICO (77.8%: n=7/9pts vs. 20.0%: n=2/10pts, p < 0.05). No patients with retrograde flow of M1 showed the retrograde flow at A1 or OphA (p < 0.05).

## Conclusions

4D-Flow is valuable for depicting the complicated flow at M1, A1, PCoA and OphA. Abnormally retrograde flow at M1 was related to the pathological condition before surgery and the flow direction at A1 and OphA. 4D-Flow can visualize the complementary flow from circles of Willis, OphA and donor artery.

**Figure 1:**
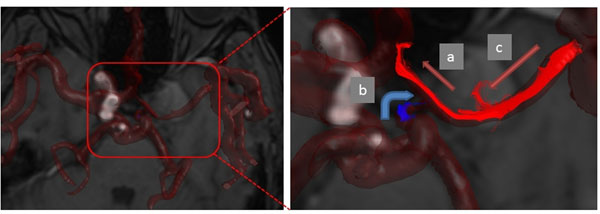
The case with IC aneurysm post ligation of ICA and EC-IC bypass. Left: Whole cerebral arteries image. Right: a: retrograde A1 flow; b: anterograde PCoA flow; c: retrograde M1 flow

**Figure 2:**
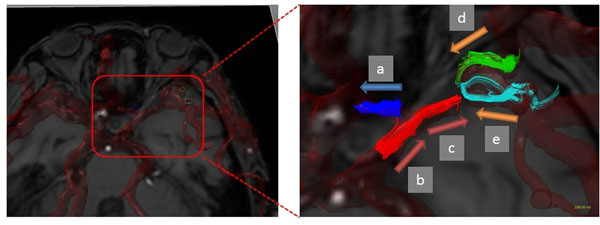
The case with ICO post EC-IC bypass. Left: Whole cerebral arteries image. Right: a: anterograde A1 flow; b: retrograde PCoA flow; c: anterograde M1 flow; d,e: flow via EC bypass.

